# Estimating Primary Production of Picophytoplankton Using the Carbon-Based Ocean Productivity Model: A Preliminary Study

**DOI:** 10.3389/fmicb.2017.01926

**Published:** 2017-10-05

**Authors:** Yantao Liang, Yongyu Zhang, Nannan Wang, Tingwei Luo, Yao Zhang, Richard B. Rivkin

**Affiliations:** ^1^Research Center for Marine Biology and Carbon Sequestration, Shandong Provincial Key Laboratory of Energy Genetics, Qingdao Institute of Bioenergy and Bioprocess Technology, Chinese Academy of Sciences, Qingdao, China; ^2^Institute of Marine Microbes and Ecospheres, State Key Laboratory of Marine Environmental Science, Xiamen University, Xiamen, China; ^3^Department of Ocean Sciences, Memorial University of Newfoundland, St. John’s, NL, Canada

**Keywords:** carbon-based production model, abundance, growth rate, primary production, picophytoplankton

## Abstract

Picophytoplankton are acknowledged to contribute significantly to primary production (PP) in the ocean while now the method to measure PP of picophytoplankton (PP_Pico_) at large scales is not yet well established. Although the traditional ^14^C method and new technologies based on the use of stable isotopes (e.g., ^13^C) can be employed to accurately measure *in situ* PP_Pico_, the time-consuming and labor-intensive shortage of these methods constrain their application in a survey on large spatiotemporal scales. To overcome this shortage, a modified carbon-based ocean productivity model (CbPM) is proposed for estimating the PP_Pico_ whose principle is based on the group-specific abundance, cellular carbon conversion factor (CCF), and temperature-derived growth rate of picophytoplankton. Comparative analysis showed that the estimated PP_Pico_ using CbPM method is significantly and positively related (*r*^2^ = 0.53, *P* < 0.001, *n* = 171) to the measured ^14^C uptake. This significant relationship suggests that CbPM has the potential to estimate the PP_Pico_ over large spatial and temporal scales. Currently this model application may be limited by the use of invariant cellular CCF and the relatively small data sets to validate the model which may introduce some uncertainties and biases. Model performance will be improved by the use of variable conversion factors and the larger data sets representing diverse growth conditions. Finally, we apply the CbPM-based model on the collected data during four cruises in the Bohai Sea in 2005. Model-estimated PP_Pico_ ranged from 0.1 to 11.9, 29.9 to 432.8, 5.5 to 214.9, and 2.4 to 65.8 mg C m^-2^ d^-1^ during March, June, September, and December, respectively. This study shed light on the estimation of global PP_Pico_ using carbon-based production model.

## Introduction

Marine picophytoplankton, which mainly include the autotrophic *Prochlorococcus, Synechococcus*, and picoeukaryotes, are autotrophic prokaryotes and eukaryotes with an equivalent spherical diameter of less than 2–3 μm. Their abundance and distributions in the ocean have been well-studied during the past two decades. Now it is well known that picophytoplankton are ubiquitous and abundant (i.e., 10^2^ to 10^6^ cells mL^-1^) in the photic zone and contribute significantly to autotrophic carbon biomass and primary production (PP) (Worden et al., 2004, 2015; Jardillier et al., 2010; Buitenhuis et al., 2012). In some oligotrophic regions, this diverse group can contribute up to 80% of the fixed carbon in the ocean (Campbell et al., 1994; Partensky and Garczarek, 2010). Among picophytoplankton, *Prochlorococcus* is abundant (up to 10^6^ cells mL^-1^) in the ocean at a wide latitudinal range, i.e., 45°N to 40°S (Scanlan et al., 2009), and are particular abundant in oligotrophic areas (Partensky and Garczarek, 2010). In comparison with *Prochlorococcus*, abundances of *Synechococcus* are generally one to two orders of magnitude lower, they are more widely distributed in the ocean and usually most abundant in mesotrophic seawaters (Partensky et al., 1999; Zhang et al., 2008; Cottrell and Kirchman, 2009). Picoeukaryotes are much less abundant than *Prochlorococcus* and *Synechococcus* in the ocean, while they are as important in terms of biomass and PP as picocyanobacteria (Worden et al., 2004, 2015; Jardillier et al., 2010; Buitenhuis et al., 2012).

Although picophytoplankton are acknowledged to contribute very importantly to oceanic PP, whereas so far the accurate estimation of the PP of picophytoplankton (PP_Pico_) in a wide survey on large spatiotemporal scales is still challenging. This is due to the traditional ^14^C method to measure *in situ* PP_Pico_ is much time-consuming and labor-intensive, which constrains its actual application in global surveys. In addition to the traditional ^14^C method, the new technologies (e.g., NanoSIMS) based on the uptake of natural abundances of the stable isotopes (e.g., ^13^C) have open new perspectives in the measurement of the *in situ* phytoplanktonic CO_2_ fixation (Popa et al., 2007; Ploug et al., 2010; Klawonn et al., 2016). The *in situ* measurement of PP_Pico_ using the new technologies could enhance our understanding and provide new data about PP_Pico_. So far, our understanding of picophytoplankton PP_Pico_ is much more limited than their global distributions and diversity. This paucity of data also limits our in-depth understanding about their contributions to ocean carbon cycles (Jiao et al., 2010). To reduce the gaps in knowledge about the PP_Pico_ at large spatial and temporal scale, the development of accurate prediction model is considered as a promising approach to evaluate the PP_Pico_. The PP of total phytoplankton in the global ocean had been well studied by using model predictions (Behrenfeld and Falkowski, 1997; Field et al., 1998; Tilstone et al., 2015), whereas the relative contribution of picophytoplankton among the total phytoplankton to the oceanic PP is not well understood. Recently, a pigment-based modeling of PP was applied to estimate the size-dependent PP using the remotely sensed chlorophyll (Chl) concentration (Uitz et al., 2008, 2010, 2012; Kheireddine et al., 2017). However, the relationship between Chl and carbon biomass (C) of phytoplankton in response to the variability of light, nutrient stress, taxonomy, and other environmental stressors is extremely plastic (Geider, 1987; Falkowski and La Roche, 1991), also the PP refers to the rate of carbon turnover, but not Chl, therefore carbon biomass rather than Chl is more appropriate to describe the standing stocks of picophytoplankton, and is more suitable to estimate the PP (Westberry et al., 2008). Moreover, the carbon biomass of picophytoplankton appears to be well related with their abundance (Buitenhuis et al., 2012), whereas the relationship between PP and abundance of picophytoplankton has not yet been well established.

In this study, an adaptation of the carbon-based production model (CbPM) of Behrenfeld et al. (2005) was proposed to estimate the PP of specific groups of picophytoplankton, e.g., *Prochlorococcus, Synechococcus*, and picoeukaryotes. The rates of carbon production of the three abundant and important marine autotrophic picoplankton can be estimated from the following parameters, literature-reported carbon conversion factors (CCF), temperature dependent growth rates, *in situ* cell abundances of picophytoplankton and remotely determined environmental variables. Defining the relationship between PP and picophytoplankton abundance will contribute to the development of a modeling method for estimating the PP_Pico_. Future application of the CbPM for large-scale investigation of the PP_Pico_ will contribute to a deeper understanding of the important contributions of picophytoplankton to the marine carbon cycle in the global oceans.

## Materials and Methods

### Modeling Primary Production of Picophytoplankton

PP of *Prochlorococcus, Synechococcus*, and picoeukaryotes was estimated from a modification of the carbon-based PP model of Behrenfeld et al. (2005).

(1) $$\begin{array}{c} \mathrm{PP}\;=\;C\;\times \;\mu \;\times \;Z_{\mathrm{eu}}\;\times \;h(I_{0}) \end{array}$$

where PP is the depth integrated primary production (mg C m^-2^ d^-1^), C is the carbon biomass of picophytoplankton in the surface layer (mg C m^-3^), μ is the growth rate (d^-1^), *Z*_eu_ is the depth of euphotic zone (m), and *h*(*I*_0_) describes how changes in surface irradiance influence the depth-dependent profile of carbon fixation.

The C of picophytoplankton was computed as the product of cell abundance and cellular carbon content using published CCF. The minimum, maximum, and average values of CCF of unialgal cultures for *Prochlorococcus, Synechococcus*, and picoeukaryotes were compiled by Buitenhuis et al. (2012) and shown in **Table 1**. In this study, the average CCF of 36, 255, and 2590 fg C cell^-1^ for *Prochlorococcus, Synechococcus*, and picoeukaryotes, respectively, were used to calculate the group-specific picophytoplankton biomass.

**Table 1:** Carbon conversion factors as reported by Buitenhuis et al. (2012). Here, we used the average value.

	Carbon conversion factors (fg C cell^-1^)		
	Min	Max	Average
*Prochlorococcus*	16	53	36
*Synechococcus*	170	350	255
Picoeukaryotes	800	4400	2590

The temperature-dependent growth rates of *Prochlorococcus, Synechococcus*, and picoeukaryotes were estimated from published growth–temperature relationships (Johnson et al., 2006; Chen et al., 2014; Pittera et al., 2014). Binominal equation was used to describe the temperature dependence of growth rate of *Prochlorococcus*, as their relationships were not linear and not suitable for the application of Arrhenius equation. An Arrhenius equation was used to describe the temperature dependence of growth rate of *Synechococcus* and picoeukaryotes, μ = μ_c_e*^-E/kT^*, in which μ is the growth rate varying with temperature, μ_c_ is a normalization constant, *E* is the activation energy (eV, 1 eV = 96.49 kJ mol^-1^), *k* is the Boltzmann constant (8.62 × 10^-5^ eV K^-1^), and *T* is absolute temperature (*K*) (Brown et al., 2004).

According to the original model (Behrenfeld et al., 2005), *Z*_eu_ was calculated as:

(2) $$\begin{array}{c} Z_{\mathrm{eu}}\;=\;\ln(0.01)/k490 \end{array}$$

The *h*(*I*_0_) is computed as:

(3) $$\begin{array}{c} {h(I}_{0}) = 0{.66125 I}_{0}{/(I}_{0}+ 4.1) \end{array}$$

As *Z*_eu_ in the original model was developed for oligotrophic and upwelling waters and may overestimate the *Z*_eu_ in the turbid coastal waters (Shang et al., 2011; Tripathy et al., 2012), the MODIS/Aqua *Z*_eu_ products based on inherent optical properties (IOP-approach) (Lee et al., 2005, 2007; Shang et al., 2011) was used in the Bohai Sea ^1^.

The PPs of *Prochlorococcus, Synechococcus*, and picoeukaryotes were calculated according to the Eqs 1–3. The PP_Pico_ is the sum of PPs of *Prochlorococcus, Synechococcus*, and picoeukaryotes.

### Data on Primary Production and Abundance of Picophytoplankton

**FIGURE 1:**
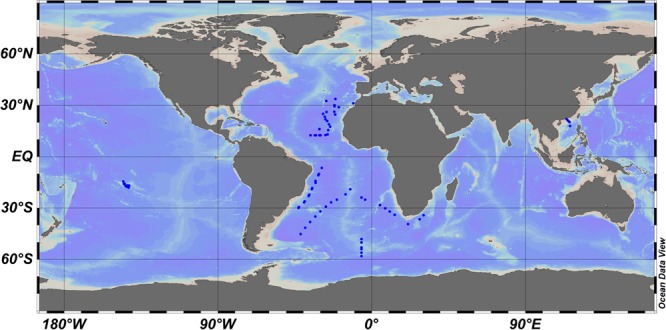
Location of the stations where the abundance and ^14^C-based primary production of picophytoplankton were measured.

To test the reliability and validity of the modified CbPM method, we compared the estimated PP_Pico_ by CbPM with the actually measured PP_Pico_ data using the radiolabeled carbon uptake method (i.e., ^14^C method). Firstly, for this purpose, a field dataset of PP and abundance of picophytoplankton (**Figure 1** and **Supplementary Data Sheet 1**) was compiled from Atlantic Meridional Transect (Marañón et al., 2003), Southern Ocean (Smetacek et al., 1997), Atlantic Ocean (Li, 1994; Jardillier et al., 2010; Hartmann et al., 2014), South China Sea (Chen et al., 2014; Xie and Huang, unpublished data), and French Polynesian atoll lagoons (Charpy and Blanchot, 1998). In this field datasets, the PP_Pico_ was measured using the ^14^C uptake method, and the abundance of picophytoplankton were measured using flow cytometry.

### Picophytoplankton Abundance and Environmental Variables in the Bohai Sea, China

A case study and test of concept of the modified CbPM was conducted in the Bohai Sea, China to estimate the PP_Pico_. The Bohai Sea is a large semi-enclosed shallow sea basin in the western Pacific Ocean (117° 30′–121°E, 37–41°N), with an average depth of 18.7 m (Zhang et al., 2004). It includes three coastal bays (Liaodong, Bohai, and Laizhou Bays) and central Bohai Sea. Summers are wet and warm. Winters are cold and dry, with strong northerly monsoons blowing from late November to March. Spring and autumn are transitional seasons between summer and winter (Tang, 2003). The Bohai Sea in general has been extensively studied since the late 1950s (Zhang et al., 2004). Although the distributions of phytoplankton, Chl *a*, PP, and nutrients are well defined (Sun et al., 2002; Wei et al., 2004; Zhang et al., 2004), little is known about the PP_Pico_ in the Bohai Sea.

Four field expeditions were conducted during 2005 (March 26 to April 12, June 10 to July 11, September 9–24, and November 30 to December 8) in the Bohai Sea. During December, samples were collected only from the surface layer of the western areas. During the other sampling periods, when the water depth was less than 10 m, samples were collected only from the surface layer; when the water depth was between 10 and 20 m, samples were collected from the surface, 5 and 10 m layers; and when the water depth was deeper than 20 m, water samples were collected from the surface, 10 and 20 m layers using 10-L Niskin bottles.

Triplicate 2 mL water samples were collected from Niskin bottles mounted on a Rosette sampling assembly and were fixed on-board to a final concentration of 1% glutaraldehyde. After 15–20 min of fixation in dark at room temperature, samples were immersed in liquid nitrogen for 10 min and then stored in -80°C until further analysis. Picophytoplankton were analyzed on an Epics Altra II flow cytometer (Beckman Coulter, United States) with a 306C–5 argon laser (Coherent, United States) according to Jiao et al. (2002).

Environmental variables of Bohai Sea required for estimating PP_Pico_ were compiled from the monthly average Level-3 4-km MODIS/Aqua data. These include sea surface temperature (°C), the depth of euphotic zone (*Z*_eu_; m), diffuse attention coefficients at 490 nm (k490: m^-1^), surface Chl (mg m^-3^), and surface photosynthesis active radiation (*I*_0_; moles photons m^-2^ h^-1^) for the corresponding sampling stations from March to December 2005. Data were downloaded from the NASA Ocean Color website (see text footnote 1). The temperature profile data of Bohai Sea was extracted from the World Ocean Atlas 2013 (Ocean Data View website ^2^) and the resolution was 0.25° × 0.25° grids.

### Statistical and Sensitivity Analysis

Analysis of variance (ANOVA) was used to assess differences in picophytoplankton abundances at different depth during each expedition (SPSS 18) and Model 2 regression (Reduced Major Axis) was used to assess the relationships between selected parameters (Ricker, 1973; Blackburn and Gaston, 1998).

## Results and Discussion

### Estimation of the Growth Rates of Picophytoplankton

**FIGURE 2:**
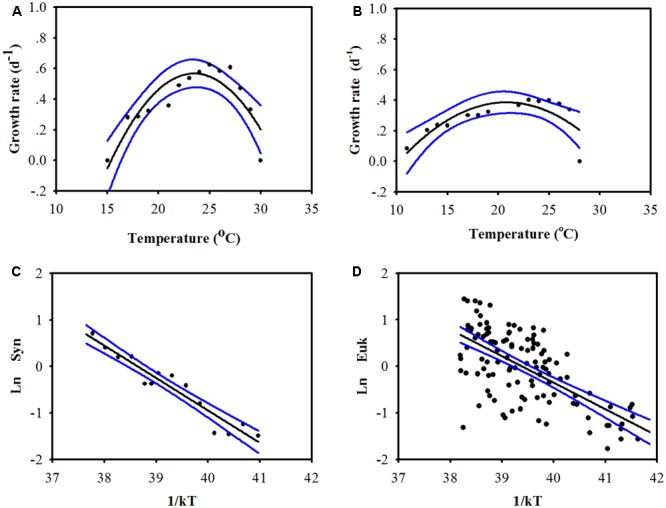
Relationship between temperature and the growth rates of low-latitude-dominated *Prochlorococcus* (**A**, eMIT9312, using lab data, growth rate = –4.17 + 0.40 × temperature – 0.0086 × temperature^2^, *r*^2^ = 0.78, *P* < 0.001), high-latitude-dominated *Prochlorococcus* (**B**, eMED4, using lab data, growth rate = –1.11 + 0.14 × temperature – 0.0035 × temperature^2^, *r*^2^ = 0.60, *P* < 0.01), *Synechococcus* [**C**, using lab data, according to the Arrhenius equation, LnμSyn = –0.73 (CI = –0.88 to –0.60)/*kT* + 28.13 (CI = 23.20–34.15), *r*^2^ = 0.92, *P* < 0.001], and picoeukaryotes [**D**, using field data when Chl is higher than 0.5 mg m^-3^, according to the Arrhenius equation, LnμEuk = –0.86 (CI = –1.07 to –0.68)/*kT* + 33.85 (CI = 26.94–42.35), *r*^2^ = 0.41, *P* < 0.001]. The blue lines and the value in the parentheses represent the 95% confidence interval (CI). The growth rate data of *Prochlorococcus, Synechococcus*, and picoeukaryotes were collected from Johnson et al. (2006), Chen et al. (2014), and Pittera et al. (2014), respectively.

The group-specific growth rates of the picophytoplankton community were significantly related to temperature (**Figure 2**). For *Prochlorococcus*, the laboratory-determined growth rate of the two most abundant *Prochlorococcus* ecotypes (eMIT9312 and eMED4) in tropical and temperate waters were compiled from Johnson et al. (2006) and Biller et al. (2015). Temperature was a statistically significant predictor of growth rates for both *Prochlorococcus* eMIT9312 and eMED4 (*r*^2^ = 0.78, *P* < 0.001 and 0.60, *P* < 0.01, respectively. **Figures 2A,B**). As the two high light-adapted *Prochlorococcus* ecotypes are abundant in tropical and temperate waters, the relationships between growth rate and temperature were used to estimate the growth of *Prochlorococcus* in the CbPM.

For *Synechococcus*, the average temperature-dependent growth rate of six marine *Synechococcus* strains (tropical, A15-37 and M16.1; mid-latitude, WH7803 and ROS8604 and high-latitude, MVIR-16-2 and MVIR-18-1; Pittera et al., 2014) was computed for the temperature range of 10–34°C). Temperature was appeared also as a statistically significant predictor (*r*^2^ = 0.92, *P* < 0.001; **Figure 2C**) that closely correlated with the growth rates of *Synechococcus.*

The dataset used to simulate the relationships between temperature and the growth rates of *Prochlorococcus* and *Synechococcus* was from studies that used cultured strains isolated from particular marine sites (Johnson et al., 2006; Pittera et al., 2014; Biller et al., 2015). The data from limited number strains does not represent the full range of growth characteristic of *Prochlorococcus* and *Synechococcus*, although these datasets were widely used in other modeling studies (Boyd et al., 2015; Hynes et al., 2015; Stawiarski et al., 2016; Grossowicz et al., 2017). We recognize that the high phenotypic diversity of *Prochlorococcus* and *Synechococcus* combined with the limited number of cultured strains for which there are growth rates data represents an inherent limitation of model parameterizations.

**FIGURE 3:**
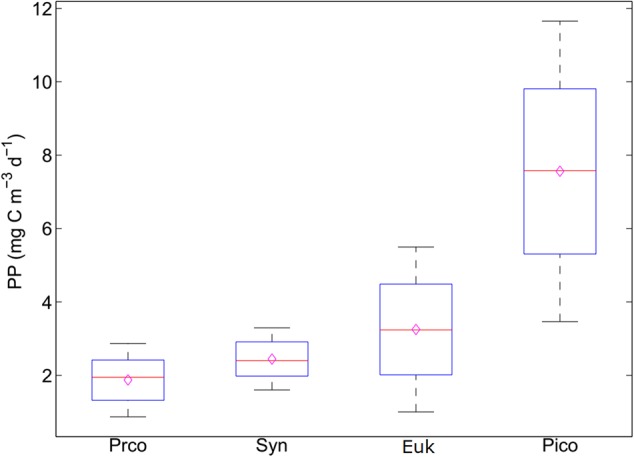
Sensitivity analysis of the carbon conversion factors on the primary production of picophytoplankton. The black lines represent the 95% confidence interval. The red line represents the median value. The hollow purple diamond represents the mean value. Proc, *Prochlorococcus*; Syn, *Synechococcus*; Euk, picoeukaryotes; Pico, picophytoplankton.

While the prokaryotic fraction of picophytoplankton is dominated by two genera, *Prochlorococcus* and *Synechococcus*, the picoeukaryotic fraction is much more diverse and nearly every algal classes contain its representative species (Vaulot et al., 2008; de Vargas et al., 2015; Worden et al., 2015). Hence, the temperature-dependent growth rate of single picoeukaryotic taxa would not have been suitable for estimating growth rate of total picoeukaryotic community. Therefore, the temperature-dependent growth rate of picoeukaryotes was estimated using the field-measured growth rates of picoeukaryotic community reported by Chen et al. (2014). When the total Chl concentration is higher than 0.5 mg m^-3^, the growth rates of picoeukaryotes were related with temperature (*r*^2^ = 0.41, *P* < 0.001; **Figure 2D**). However, when the total Chl concentration is less than 0.5 mg m^-3^, the growth rates of picoeukaryotes were not significantly related with temperature (*P* > 0.05). The variability in the relationship between growth rates of picoeukaryotes and temperature is relatively large (**Figure 2D**), suggests that environmental factors which were not included in our model (e.g., light intensity, nutrients, Chl concentration, etc.) were important for the growth rates of picoeukaryotes (Chen et al., 2014). Based on the analyses presented in **Figure 2D**, the model may overestimate the growth rate of picoeukaryotes by an average of 58%. Picoeukaryotes represent variable fraction of the total picophytoplankton community (**Table 2**) and contribution to their photosynthetic carbon production (**Figure 3**). Hence, the proposed model introduces a level of uncertainty to the estimation of PP_Pico_. More field data about the relationship between temperature and the growth rates of picoeukaryotes and the integration of other environmental factors into the estimation of the growth rates of picoeukaryotes would help to improve the accuracy of the model estimates.

**Table 2:** Mean and standard deviation of surface environmental parameters and abundance, carbon biomass, and primary production of picophytoplankton in the Bohai Sea.

	March^a^	June^a^	September^a^	December^a^
Temperature (°C)	5.9 ± 2.3	21.1 ± 3.5	23.6 ± 0.6	6.1 ± 1.0
Chlorophyll (mg m^-3^)	4.4 ± 1.3	4.7 ± 1.3	5.4 ± 1.9	4.2 ± 1.2
*I*_0_ (mol photons m^-2^ d^-1^)	40.4 ± 2.0	49.3 ± 2.0	35.5 ± 1.6	16.2 ± 0.4
*Z*_eu_ (m)	8.9 ± 4.3	15.1 ± 4.6	9.3 ± 3.2	6.7 ± 3.0
k490 (m^-1^)	0.3 ± 0.1	0.3 ± 0.2	0.4 ± 0.1	0.3 ± 0.01
Syn (10^4^ cells mL^-1^)	0.15 ± 0.1	2.2 ± 2.0	1.4 ± 1.0	2.3 ± 1.3
Euk (10^3^ cells mL^-1^)	1.1 ± 1.3	4.8 ± 6.8	3.1 ± 2.4	5.7 ± 4.8
Biomass_Syn_ (mg C m^-3^)^b^	0.4 ± 0.3	5.7 ± 5.1	3.6 ± 2.5	6.0 ± 3.3
Biomass_Euk_ (mg C m^-3^)^c^	2.8 ± 3.5	12.4 ± 17.6	8.0 ± 6.2	14.8 ± 12.3
μSyn (d^-1^)	0.11 ± 0.02	0.55 ± 0.19	0.66 ± 0.04	0.11 ± 0.01
μEuk (d^-1^)	0.15 ± 0.04	1.01 ± 0.42	1.24 ± 0.10	0.15 ± 0.02
PP_Syn_ (mg C m^-2^ d^-1^)^b^	0.2 ± 0.1	35.1 ± 38.2	11.6 ± 8.4	2.7 ± 1.9
PP_Euk_ (mg C m^-2^ d^-1^)^c^	3.4 ± 3.8	76.3 ± 109.1	40.2 ± 46.6	15.2 ± 15.4
PP_Pico_ (mg C m^-2^ d^-1^)^d^	3.6 ± 3.9	111.4 ± 106.5	51.8 ± 52.4	17.9 ± 17.0

*^a^The four cruises were conducted from March 26 to April 12, June 10 to July 11, September 9–24, and November 30 to December 8, 2005. ^b^The average carbon conversion factor for *Synechococcus* cultures is 255 fg C cell^-1^ (Buitenhuis et al., 2012). ^c^The average carbon conversion factor for picoeukaryotes cultures is 2590 fg C cell^-*1*^ (Buitenhuis et al., 2012). ^d^PP_*Pico*_ = PP_*Syn*_ + PP_*Euk*_. *I*_*0*_, surface photosynthesis active radiation; k490, diffuse attention coefficients at 490 nm; Syn, *Synechococcus*; Euk, picoeukaryotes; μ, growth rate; PP, primary production; Pico, picophytoplankton.*

### The Influence of CCF on the Calculation of PP_Pico_

To test the influence of CCF on the calculated PP_Pico_, the PP_Pico_ is calculated according to Eq. 4 (PP = C × μ), in which C is the product of the measured cell abundances and CCF values. The CCFs for each functional type of picophytoplankton is selected from the minimum to the maximum at increments of 10% (Buitenhuis et al., 2012). Sensitivity analysis showed that there is some uncertainty in the CCF, with larger variations for picoeukaryotes (∼5-fold) than *Prochlorococcus* and *Synechococcus* (two- to threefold), and this can lead to a ∼3-fold variation in computed PP_Pico_ (**Figure 3**). It follows that the determination of appropriate CCF is essential for accurate estimation of the picophytoplankton biomass and production. Besides the use a fixed CCF, the CCF could be calculated from cell size or volume (Buitenhuis et al., 2012). However, the cell size of different phytoplanktonic group could not be separated by the traditional flow cytometry method (Jiao et al., 2002; Buitenhuis et al., 2012). In future, the applications of new technologies (e.g., multi-laser flow cytometry method and the combination of fluorescence in situ hybridization and flow cytometry, etc.) into the measurement of calibrated cell size of particular picophytoplankton group could improve the accurate estimation of the picophytoplankton biomass (Thompson and van den Engh, 2016; Riou et al., 2017).

Buitenhuis et al. (2012) compiled the CCF from both unialgal cultures and *in situ* samples. The *in situ* CCF was calculated from the cell sizes estimated from flow cytometry and carbon:volume relationships (Campbell et al., 1994; Garrison et al., 2000; Worden et al., 2004; Bec et al., 2008). Due to the large variability of ratio of cell carbon:volume of picoplankton, the use of cell volume does not provide a clear advantage over CCF to estimate carbon biomass. Buitenhuis et al. (2012) compared the influence of the CCF directly measured and *in situ* estimated (the average values were 60, 154, and 1319 fg C cell^-1^ for *Prochlorococcus, Synechococcus*, and picoeukaryotes, respectively) on the estimation of carbon biomass using they compiled global picophytoplankton abundance dataset. Their analyses showed that the average carbon biomass using the *in situ* CCFs is 72% of that estimated using from that directly measured. Using the dataset in this study (**Supplementary Data Sheet 1**), we compared the influence of the direct and *in situ* CCFs on the estimation of carbon biomass and PP_Pico_. The estimated carbon biomass using the direct and *in situ* CCFs was 10.0 ± 10.8 and 10.0 ± 7.7 mg C m^-3^ (*n* = 171), respectively, and the estimated PP_Pico_ was 7.3 ± 11.6 and 5.2 ± 6.9 mg C m^-3^ d^-1^, respectively. Although the differences in cell carbon content in laboratory grown and *in situ* populations could introduce uncertainties in the estimation of carbon biomass and PP_Pico_, other well-accepted models and modeling studies used these CCFs to represent *in situ* processes (Buitenhuis et al., 2012). In future, the routine measurement of calibrated cell size of particular picophytoplankton group as the additional measurement was strongly recommended and could improve the accurate estimation of the picophytoplankton biomass and production (Thompson and van den Engh, 2016; Riou et al., 2017).

### Comparison of the Measured and Model-Estimated Primary Production of Picophytoplankton

Model-estimated and measured PP_Pico_ were compared using the CbPM model based on the datasets of picophytoplankton abundance and their concomitantly measured PP using the ^14^C-uptake method. The data sets represent a wide geographic area and ocean domains and include the Atlantic Meridional Transect (Marañón et al., 2003), Southern Ocean (Smetacek et al., 1997), Atlantic Ocean (Li, 1994; Jardillier et al., 2010; Hartmann et al., 2014), South China Sea (Chen et al., 2014; Y. Xie and B. Huang, unpublished data), and French Polynesian atoll lagoons (Charpy and Blanchot, 1998) (**Figure 1** and **Supplementary Data Sheet 1**).

**FIGURE 4:**
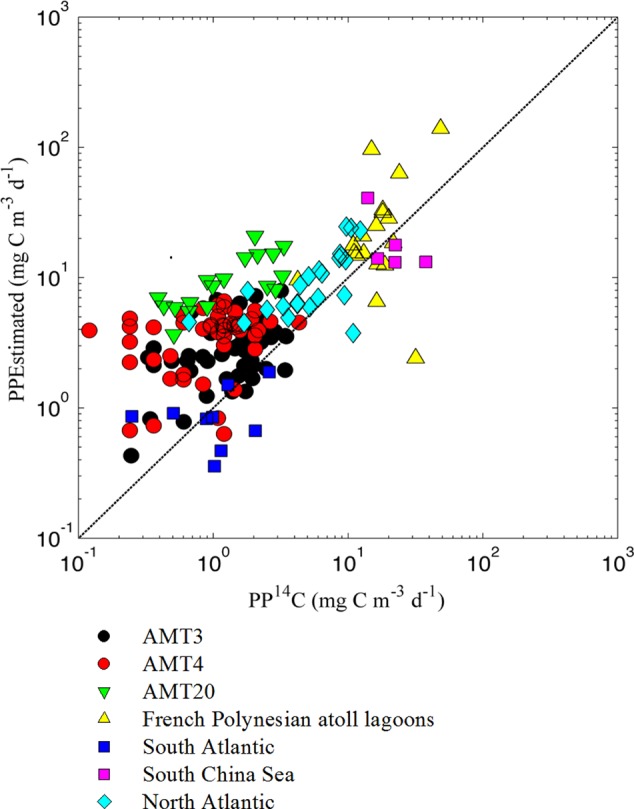
Relationship between model calculated and ^14^C-based estimates of primary production conducted in different ocean regions [PP_Estimated_ = 1.73 (CI = 1.49–1.99) × PP^14^C + 0.57 (CI = 0.36–1.63), *r*^2^ = 0.53, *P* < 0.001, *n* = 171, Model 2 regression, Reduced Major Axis]. Calculated primary production was obtained by multiplying picophytoplankton growth rates times picophytoplankton carbon biomass. Carbon conversion was obtained from abundances of the picophytoplanktonic community and established average carbon to abundance conversions (Buitenhuis et al., 2012). The ^14^C-based primary production data were collected from Atlantic Meridional Transect (Marañón et al., 2003), Southern Ocean (Smetacek et al., 1997), Atlantic Ocean (Li, 1994; Jardillier et al., 2010; Hartmann et al., 2014), South China Sea (Chen et al., 2014; Y. Xie and B. Huang, unpublished data), and French Polynesian atoll lagoons (Charpy and Blanchot, 1998). The dashed black line represents the 1:1 line.

The computed PP_Pico_ (sum of PPs of *Prochlorococcus, Synechococcus*, and picoeukaryotes), ranged from 0.04 to 104.8 mg C m^-3^ d^-1^, and the estimated and measured PP_Pico_ were significantly related (*r^2^* = 0.53 and 0.46 for normal and log_10_-transformed data, respectively, *P* < 0.001, *n* = 171; **Figure 4**). This suggested the practical applicability of CbPM to estimate the PP_Pico_. A Model 2 regression was used to assess the relationship between directly measured PP_Pico_ (using size fractionated ^14^C uptake) and model predicted PP_Pico_. The slope of the relationship was greater than 1 (i.e., slope = 1.73, CI = 1.49–1.99), suggesting that our model overestimated PP_Pico_ by an average of 73% comparing to the measured PP_Pico_. This overestimation depends on the relative composition of the picophytoplankton as well as the model’s representation of their growth characteristics. The use of variable CCFs and the larger data sets representing diverse community and growth conditions will improve the future model performance.

PP of total phytoplankton community is well characterized in the global ocean (Behrenfeld and Falkowski, 1997; Field et al., 1998; Tilstone et al., 2015). However, the contribution of picophytoplankton to total PP is still poorly understood. This is because that the ratio of *in situ* PP_Pico_ to total PP using the ^14^C-uptake method is extensively time-consuming and labor-intensive (Uitz et al., 2010; Landry et al., 2011; Laws, 2013). Although pigment-based modeling of PP has been applied to estimate the size-dependent PP (Uitz et al., 2008, 2010, 2012), due to the plasticity of Chl:C in response to the variability of environmental parameters, C rather than Chl is considered more suitable to estimate the PP (Westberry et al., 2008). Moreover, since the picoeukaryotes among picophytoplankton could not be separated from nano- and micro-phytoplanktonic eukaryotes through pigment analysis, the unique contribution of picoeukaryotes to total PP of phytoplankton is hard to be characterized, despite that the contribution of picoeukaryotes could be comparable to picocyanobacteria in some marine environments (Worden et al., 2004, 2015; Jardillier et al., 2010; Uitz et al., 2010). The modified CbPM in this study provides a carbon-based protocol which also takes into consideration the contribution of picoeukaryotes for PP_Pico_ estimation. As compared to the ^14^C-uptake method, due to the simplicity and convenience, CbPM can likely become a promising substitute method for large-scale survey for PP_Pico_ estimation in future.

### Case Study of Estimating the PP_Pico_ in Bohai Sea by Using CbPM

The PP_Pico_ model was applied in the Bohai Sea, China, using the *in situ* picophytoplankton abundance and remotely sensed environmental variables. A total of 131 picophytoplankton abundance samples were collected during four seasonal 2005 cruises. *Synechococcus* and picoeukaryotes were identified and enumerated using flow cytometry (Jiao et al., 2002). *Prochlorococcus* was not detected in all samples. Previous studies showed that although *Prochlorococcus* was detected in the offshore waters of East China Sea and South China Sea, they were not detected in the Yellow Sea and Bohai Sea (Jiao and Yang, 2002; Jiao et al., 2002, 2005; Bai et al., 2012; Guo et al., 2014).

**FIGURE 5:**
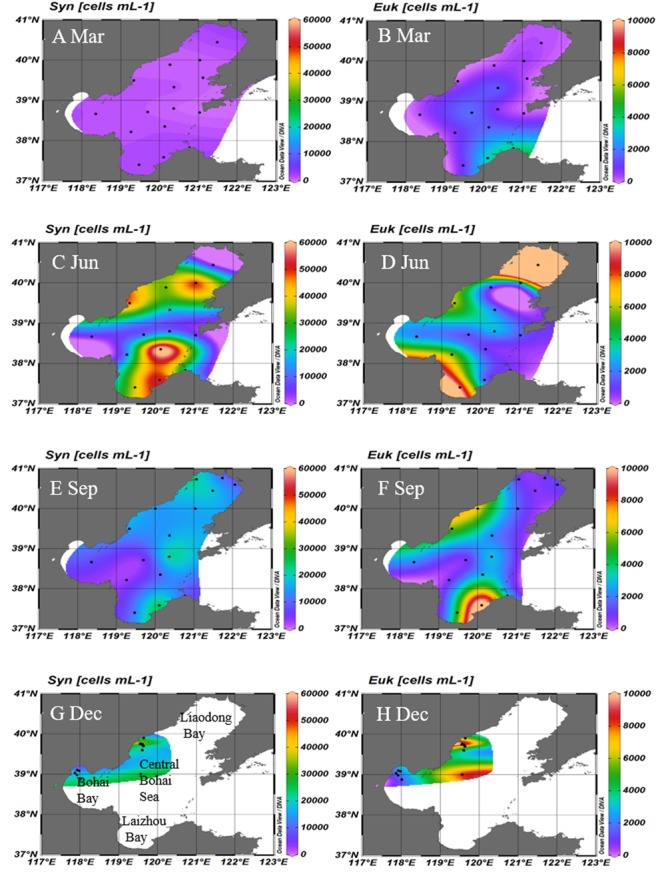
Surface distributions of *Synechococcus* **(A,C,E,G)** and picoeukaryotes **(B,D,F,H)** in the Bohai Sea in March, June, September, and December, respectively. Unit: cells mL^-1^. Black dotes represents the stations where samples were collected. Syn, *Synechococcus*; Euk, picoeukaryotes; Mar, March; Jun, June; Sep, September; Dec, December.

The abundance and distributions of *Synechococcus* and picoeukaryotes were shown in **Table 2** and **Figure 5**. During March 2005, the abundances and distributions of *Synechococcus* and picoeukaryotes were similar and higher in the southern region than northern regions (**Figures 5A,B**). During June, the abundances of *Synechococcus* were higher in Laizhou Bay and Liaodong Bay mouth (**Figure 5C**). Picoeukaryotes abundance was higher in the Liaodong and Laizhou Bays (**Figure 5D**). During September, *Synechococcus* and picoeukaryotic abundance were generally higher along the eastern and north regions of the Bohai Sea (**Figures 5E,F**). During December, *Synechococcus* and picoeukaryotic abundance were generally higher in the offshore areas of the western areas of the Bohai Sea (**Figure 5G**). No significant depth-dependent variation in the abundance of picophytoplankton was observed during any of the cruises (ANOVA, *P* > 0.05; **Supplementary Figures S1A–C**).

**Table 2** and **Supplementary Figures S2, S3** present the environmental variables in the Bohai Sea. The temperature increased from March to September (**Supplementary Figure S2** and **Table 2**) and was isothermal during the March, September, and December (**Supplementary Figure S3**). Chl concentration was relatively stable and higher than 4.2 mg m^-3^ during the four cruises. *I*_0_ and *Z*_eu_ increased from December to June. k490 was relatively stable during the four cruises (**Table 2**). The equations for *Z*_eu_ in the original CbPM model were derived from the oligotrophic and upwelling waters. The application of the equations for *Z*_eu_ might overestimate the *Z*_eu_ in turbid coastal water which is a seasonal condition in the Bohai Sea, and thus overestimate the calculated PP_Pico_ using modified CbPM model (Behrenfeld et al., 2005; Shang et al., 2011; Tripathy et al., 2012). Shang et al. (2011) showed that the MODIS/Aqua *Z*_eu_ products based on IOP-approach (Lee et al., 2005, 2007) were well related with the field-measured *Z*_eu_ in the China Sea (including the coastal and shelf waters). Hence, the MODIS/Aqua *Z*_eu_ products was used in the Bohai Sea.

**FIGURE 6:**
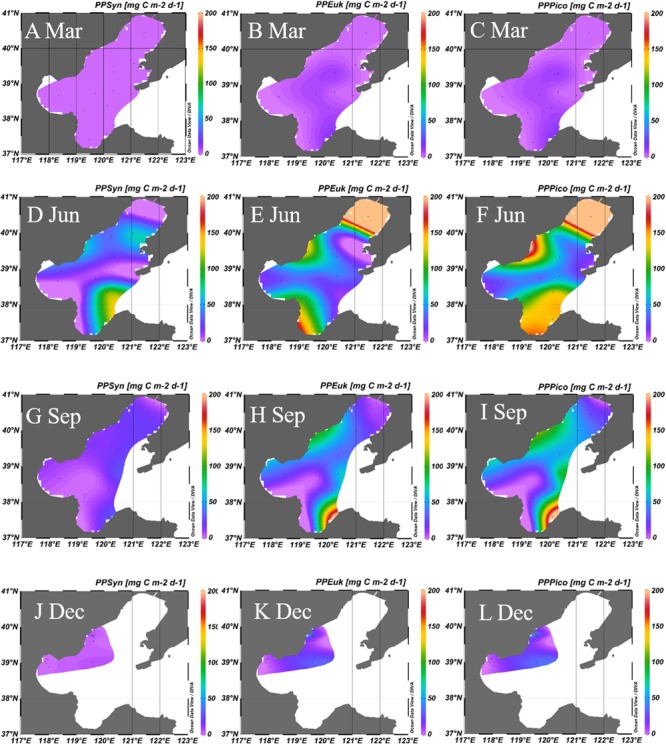
Primary production of *Synechococcus* **(A,D,G,J)**, picoeukaryotes **(B,E,H,K)**, and picophytoplankton **(C,F,I,L)** in the Bohai Sea in March, June, September, and December, respectively. Unit: mg C m^-2^ d^-1^. The carbon biomass were calculated from the average conversion factors for *Synechococcus* (255 fg C cell^-1^) and picoeukaryotes (2590 fg C cell^-1^; Buitenhuis et al., 2012). PP_Syn_, primary production of *Synechococcus*; PP_Euk_, primary production of picoeukaryotes; PP_Pico_, primary production of picophytoplankton; Mar, March; Jun, June; Sep, September; Dec, December.

The estimated PPs of *Synechococcus*, picoeukaryotes and picophytoplankton were shown in the **Table 2** and **Figure 6**. The PP_Pico_ ranged from 0.1 to 11.9, 29.9 to 432.8, 5.5 to 214.9, and 2.4 to 65.8 mg C m^-2^ d^-1^ during March, June, September, and December, respectively in Bohai Sea. The PP_Syn_, PP_Euk_, and PP_Pico_ were higher in June and September than that in March and December (**Figure 6**). Though the abundance of picoeukaryotes was lower than *Synechococcus* (**Figure 5**), since picoeukaryotes have higher CCF and growth rate as compared with *Synechococcus* (Buitenhuis et al., 2012; Chen et al., 2014), the PP_Euk_ was comparable with the PP_Syn_ (**Figure 6**), which is in accordance with the results in the Atlantic Ocean (Worden et al., 2004; Jardillier et al., 2010). In future, the simultaneously measurement of the abundance, PP and growth rates of the picophytoplankton during the field campaigns could give more information and opportunity for the improvement of the PP_Pico_ model.

## Concluding Remarks

In this study, a carbon-based PP model was employed to calculate the PP_Pico_ from the abundance and growth rates of picoplankton. The data set on global picophytoplankton abundance (Buitenhuis et al., 2012; Flombaum et al., 2013) and group-specific growth rate (Johnson et al., 2006; Chen et al., 2014; Pittera et al., 2014) could provide useful and novel information for estimating the contribution of picophytoplankton to oceanic PP. Though the modified CbPM can likely become a promising substitute method for large-scale PP_Pico_ estimation, the interpretations of the data are subject to some constraints. For example, the growth rate of a natural phytoplankton community is a function of light, nutrients, and temperature (Behrenfeld et al., 2005). In this study, the estimation of the growth rate of picophytoplankton did not consider the effects of light and nutrients. The integration of light and nutrients into the estimation of growth rate of picophytoplankton would increase the accuracy of the estimation of PP_Pico_. Moreover, in this study the available field data set of PP_Pico_ which was obtained by using ^14^C method for the verification of the model is relatively small (*n* = 171). Larger field data set of PP_Pico_ is quite necessary for a better verification of the CbPM’s practical applicability in the future. In addition, the carbon biomass was calculated basing on the cell abundance and only one same CCF for each picophytoplankton group (e.g., *Prochlorococcus, Synechococcus*, and picoeukaryotes) and the relationship between the temperature and growth rate of picoeukaryotes showed large variability (*r*^2^ = 0.41), which also introduced uncertainty of the model. In the future, routine measurement of calibrated cell size and content of particular picophytoplankton group and better fitting the relationship between growth rate and temperature would be helpful to improve the accuracy of carbon biomass estimation of picophytoplankton. Meanwhile, the integration of light and nutrients into the modeling of growth rates of *Prochlorococcus, Synechococcus*, and picoeukaryotes, and further collection of field data of growth rate and PP_Pico_ would improve the predictive accuracy of estimating growth rate and PP_Pico_. In consideration of the abundances of *Prochlorococcus* and *Synechococcus* are projected to increase 29 and 14%, respectively by the end of the 21st century (Flombaum et al., 2013), the approach reported here would shed light on the prediction of how picophytoplankton productivity respond to ocean warming in the future.

## Author Contributions

YL and YyZ designed research. YL, NW, TL, YZ, and RR performed research and analyzed data. YL, YyZ, and RR wrote the paper.

## Conflict of Interest Statement

The authors declare that the research was conducted in the absence of any commercial or financial relationships that could be construed as a potential conflict of interest.

## Footnotes

1 https://oceandata.sci.gsfc.nasa.gov/MODIS-Aqua/Mapped/Monthly/4km/

2 https://www.nodc.noaa.gov/OC5/woa13/woa13data.html

## Supplementary Material

DATA SHEET S1 The field datasets of primary production and abundance of picophytoplankton.

FIGURE S1 Vertical profiles of picophytoplankton abundance during arch **(A)**, June **(B)**, and September **(C)** 2005 in the Bohai Sea.

FIGURE S2 Sea surface temperature during March **(A)**, June **(B)**, September **(C)**, and December **(D)** 2005 in the Bohai Sea (from MODIS/Aqua).

FIGURE S3 Vertical profiles of temperature during March **(A)**, June **(B)**, September **(C)**, and December **(D)** 2005 in the Bohai Sea. The data was collected from the monthly data of World Ocean Atlas 2013 with a resolution of 0.25° × 0.25°.
