# Supplementary figures and images for: Estimating Primary Production of Picophytoplankton Using the Carbon-Based Ocean Productivity Model: A Preliminary Study
Source: Front Microbiol. 2017 Oct 5;8:1926. doi: 10.3389/fmicb.2017.01926 (PMC5633608; doi:10.3389/fmicb.2017.01926)

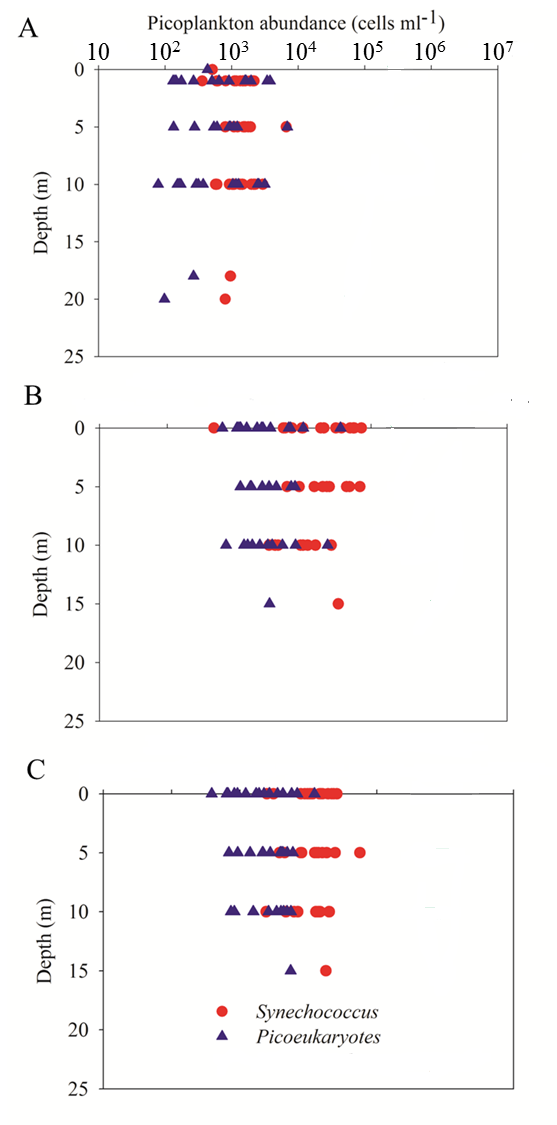

Supplement: FIGURE S1 — Vertical profiles of picophytoplankton abundance during arch (A), June (B), and September (C) 2005 in the Bohai Sea. [file Image_1.TIF]

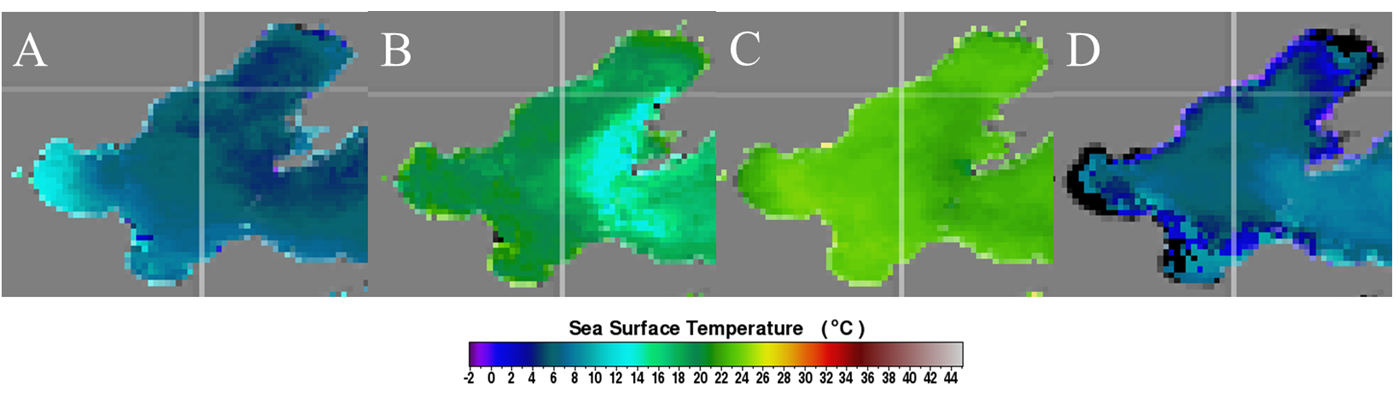

Supplement: FIGURE S2 — Sea surface temperature during March (A), June (B), September (C), and December (D) 2005 in the Bohai Sea (from MODIS/Aqua). [file Image_2.TIF]

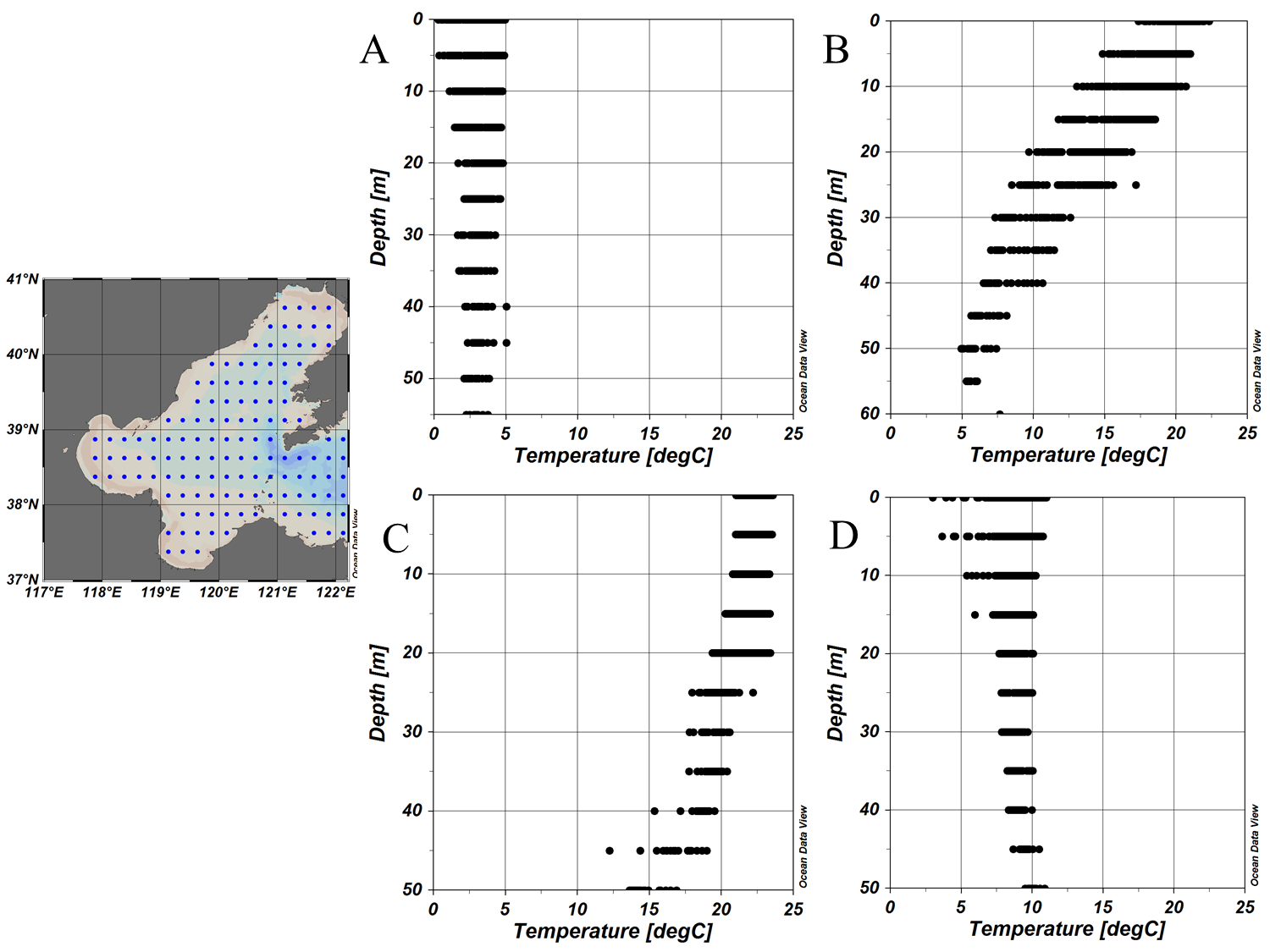

Supplement: FIGURE S3 — Vertical profiles of temperature during March (A), June (B), September (C), and December (D) 2005 in the Bohai Sea. The data was collected from the monthly data of World Ocean Atlas 2013 with a resolution of 0.25° × 0.25°. [file Image_3.TIF]
